# Deep 3D reconstruction of synchrotron X-ray computed tomography for intact lungs

**DOI:** 10.1038/s41598-023-27627-y

**Published:** 2023-01-31

**Authors:** Seungjoo Shin, Min Woo Kim, Kyong Hwan Jin, Kwang Moo Yi, Yoshiki Kohmura, Tetsuya Ishikawa, Jung Ho Je, Jaesik Park

**Affiliations:** 1 Graduate School of Artificial Intelligence, Pohang University of Science and Technology (POSTECH), Pohang, 37673 Republic of Korea; 2 School of Interdisciplinary Bioscience and Bioengineering, Pohang University of Science and Technology (POSTECH), Pohang, 37673 Republic of Korea; 3 Department of Electrical Engineering and Computer Science, Daegu Gyeongbuk Institute of Science and Technology (DGIST), Daegu, 42988 Republic of Korea; 4 Department of Computer Science, University of British Columbia (UBC), Vancouver, V6T 1Z4 Canada; 5 RIKEN SPring-8 Center, 1-1-1 Kouto, Sayo-cho, Sayo, Hyogo 679-5198 Japan; 6 Nanoblesse Research Lab., Nanoblesse, 4th fl. 85-11, Namwon-ro, Pohang, 37883 South Korea; 7 Department of Computer Science and Engineering, Pohang University of Science and Technology (POSTECH), Pohang, 37673 Republic of Korea

**Keywords:** 3-D reconstruction, X-ray tomography

## Abstract

Synchrotron X-rays can be used to obtain highly detailed images of parts of the lung. However, micro-motion artifacts induced by such as cardiac motion impede quantitative visualization of the alveoli in the lungs. This paper proposes a method that applies a neural network for synchrotron X-ray Computed Tomography (CT) data to reconstruct the high-quality 3D structure of alveoli in intact mouse lungs at expiration, without needing ground-truth data. Our approach reconstructs the spatial sequence of CT images by using a deep-image prior with interpolated input latent variables, and in this way significantly enhances the images of alveolar structure compared with the prior art. The approach successfully visualizes 3D alveolar units of intact mouse lungs at expiration and enables us to measure the diameter of the alveoli. We believe that our approach helps to accurately visualize other living organs hampered by micro-motion.

## Introduction

Pulmonary alveoli are the gas exchange units in the lungs. Gas is exchanged at the surface membranes of the alveoli by direct diffusion, and is therefore affected by their 3D morphology^1,2^. Pulmonary diseases^3,4^ that destroy alveolar morphology can hamper gas exchange and be life-threatening. However, knowledge about real alveolar morphology in intact lungs is limited^2,5^ due to the absence of a proper visualization approach.

Thoracic structure obstructs visualization of 3D alveoli in intact lungs and the visualization without thoracic opening remains a challenging task. When a thorax is opened for imaging alveoli, atmospheric pressure may affect the alveolar morphology, so the natural structure of alveoli could be changed at the micrometer scale if pressure control is not perfect^2^. Histological biopsy or scanning electron microscopy requires chemical fixation of the sample, which is known to induce distortion of the tissue^6,7^. Although Intravital microscopy (IVM)^8^ and optical coherent microscopy (OCT)^9^ are direct alveolar imaging methods, they have a limitation to be required thoracic opening.

In recent studies, synchrotron X-rays, providing higher coherence and brightness than conventional X-rays^10,11^ with a significantly higher temporal resolution, have been used for CT to visualize alveoli in intact lungs^12–14^. However, their attempts to overcome the problems are limited to manual segmentation^12^, low resolution^13,15^, and post-mortem study^14^. As well as thoracic structure, one of the major problems is motion artifacts induced by heartbeat. Recently, to overcome the cardiac motion, heartbeat-gated synchrotron X-ray imaging techniques have been performed for alveolar imaging in live lungs^16,17^. But they have been limited to low resolution^16,18^ or long-breath hold the state of the lung^17^. Thus, high quality 3D alveolar imaging in intact lungs remains a challenging problem.

Deep image prior (DIP) is a widely used^19–21^ method to solve inverse problems such as denoising, super-resolution, and inpainting in an unsupervised manner. DIP demonstrates that the inductive bias of a convolutional layer is a strong prior for the representation of image signals, and results in outstanding image recovery without ground-truth data. Recently, DIP has also shown impressive ability to reconstruct medical images, notably those obtained using positron emission tomography (PET)^22^, magnetic resonance imaging (MRI)^23^, and CT^24^. The task of 3D lung reconstruction can be addressed using 3D DIP^22^, but its resolution is restricted by the amount of GPU memory. DIP has been used for 2D CT reconstruction^24^, but the method has the drawback that it cannot fully leverage 3D information.

**Figure 1:**
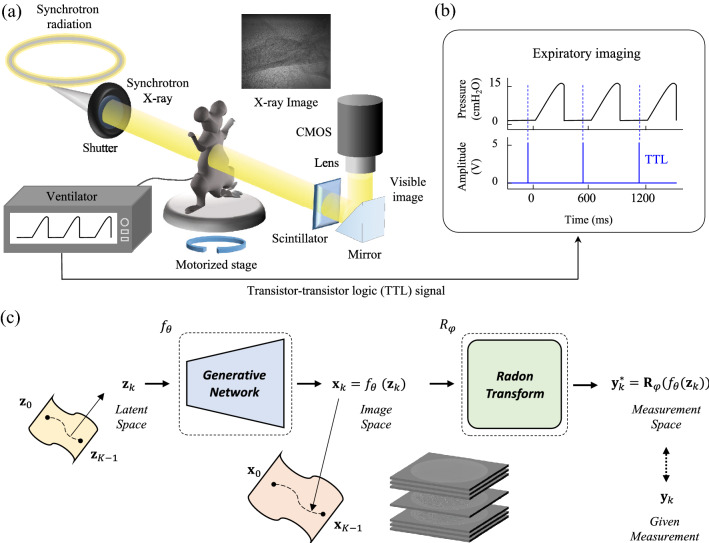
Schematic of X-ray imaging setup and framework. (**a**) A schematic of the experimental setup. (**b**) Expiratory imaging strategy during mechanical ventilation. The camera, shutter, and motorized stages are synchronized. The images are only captured at the expiration phase by TTL triggering signal fired from the mechanical ventilator during rotation of the motorized stage. (**c**) The neural network optimization steps to reconstruct computed tomography images using extended deep image prior.

For dynamic images, the latent mapping DIP method^23^ generates 2D dynamic MR images, which extended the 2D DIP framework to the time domain. Our method is closely related to^23^ in the methods of processing a sequence of 2D images. However, our latent mapping focuses on the extension to the spatial domain, which lets us consider the relationship with 2D slices to compose a 3D image, in a manner analogous to what time-domain extension does for the temporal context. In this paper, we propose a neural network algorithm for synchrotron X-ray CT reconstruction framework, capable of high-quality 3D imaging, even in the presence of motion such as breathing (Fig.1a). Importantly, we are targeting synchrotron X-ray, where extensive labeled data is difficult if not impossible to acquire. Thus, we opt for an unsupervised method based on deep image prior^19^. The technique requires only the raw synchrotron X-ray projection data that we wish to reconstruct the high-quality 3D structures; it does not require a clean image as the ground truth. For this procedure, the slicing dimension (z-axis) is encoded as a change of the latent variables describing the structure of z-slices. Importantly, sticking to 2D Neural Networks allows our method to run on a conventional GPU and still reconstruct a volume as large as ($540\times 576\times 576$) resolution.

We demonstrate new synchrotron X-ray CT images of intact lungs and the efficacy of our method by applying the approach to the task of 3D reconstruction of the alveolar structure of intact mice at expiration without opening the thorax or long breath hold. We synchronize a mechanical ventilator with our imaging system for capturing only the expiration phase of lung apex where unexpected micro-motion is minimized due to its location far from the heart even with no cardiac motion gated technique. to utilize a 2D reconstruction framework. We show that our method significantly improves imaging quality over existing alternatives.

Our results show the potential of our method, which may enable high-quality 3D imaging for various other biological applications, and thereby allow discoveries that may not have been possible due to the limitations to the use of synchrotron X-rays on live subjects such as unexpected micro-motion. We foresee the use of our method to visualize other living organs or specimens that show inevitable micro-motion.

## Method

This section introduces our approach to acquiring a new dataset for synchrotron X-ray CT for live lungs (“Synchrotron X-ray imaging”), and our approach to reconstructing the high-quality 3D shape (“Framework”).

### Synchrotron X-ray imaging

#### Animal preparation

All experimental protocols are approved by the SPring-8 Experimental Animals Care and Use Committee. This study follows ARRIVE guidelines and all methods were performed in accordance with the relevant guidelines and regulations. Total 5 Eight-week-old SPF pathogen-free nude mice (BALB/c-nu, body weight: 20–25 g, male) are anesthetized by injection of a mixture of medetomidine (1.2%, 0.3 mg/kg), butorphanol (20%, 5 mg/kg), midazolam (16%, 4 mg/kg) and saline (62.8%); the mice enter a deep sleep after about 10 min. For synchrotron X-ray imaging, a tracheostomy is performed. The mouse is mounted for the synchrotron X-ray imaging experiment, then the anesthesia is maintained using 2–3% of isoflurane in air/oxygen mixture through the endotracheal cannula by way of the mechanical ventilator (Inspira-Advanced Safety Ventilator-Pressure Controlled (ASVP), Harvard Apparatus, USA). Ventilator parameters are: respiratory rate 100 breath/min, tidal volume 10 ml/kg bodyweight, positive end-expiratory pressure 3 $\text {cmH}_{2}$O. At the end of the experiments, the mouse is euthanized by cervical dislocation under anesthesia.

#### Image acquisition

We use synchrotron X-rays to acquire projection images of a live lung by rotating the subject on a motorized stage at expiration (Fig. 1a,b). After passing through the sample, the transmitted synchrotron X-ray beam is converted by a scintillator (LSO:Tb, $5\,\textrm{mm}\times 5\,\textrm{mm}$, 8 $\upmu$m thickness) to visible light that is then reflected by a mirror (Fig. 1a). The image is magnified using an optical lens, then captured by a sCMOS (pco.edge 5.5 CLHS, PCO AG, Germany) at an effective size of 0.56 $\upmu$m and exposure time of 5 ms. To obtain the images, the lung is imaged only at the same point of the respiratory cycle (Fig. 1b) for expiratory imaging so that the lung’s position is maintained during tomography regardless of respiration. In detail, a motorized stage, shutter, and camera are synchronized and controlled by a mechanical ventilator using transistor-transistor logic (TTL) signals. As a trigger to take an image in our system, the signals are fired at the start point of every inspiration in the respiratory cycle generated by the ventilator. A total of 360 images $(2160\times 2560)$ are captured at time delays of 570 ms from the start points of inspiration in every respiratory cycle during rotation of 180$^{\circ }$ for expiratory imaging. Finally, we rescaled the images to $(540\times 640)$ having a pixel size of 2.24 $\upmu$m and cropped them to $(540\times 576)$ to adjust the tilted center of rotation (CoR). The synchrotron X-ray imaging experiments are performed at the RIKEN Coherent X-ray Optics beamline (BL29XU) at SPring-8 (http://www.spring8.or.jp), which provides high spatial and temporal resolution by using a bright monochromatic 15 keV X-ray beam (around $6\times 10^{13}$ ph/s/mm$^{2}$/mrad$^{2}$/$0.1\%$ bw). X-ray dose is below 14 Gy for a set of tomography, which is below the dose threshold (around 15 Gy^25^) for radiation-induced lung damage, thus little affecting the alveolar morphology even during 3D imaging.

### Framework

We develop a generative network to fit the measurements (the image data), an overview of our framework (Fig. 1c). We leverage the fact that we can consider 3D reconstruction as the reconstruction of stacked 2D slices. We further assume that the relationships across 2D slices of our data lay on a piece-wise linear manifold within a high-dimensional subspace: i.e., an object’s 3D structure consists of a sequence of continuous 2D slices that typically undergo smooth relative contexts. Therefore, we treat our input data as coming from a sequence of high-dimensional embeddings. This idea is similar to how^23^ dealt with time-sequence data, but with the difference that our capture sequence does not follow time and relatively smoothly varying capture conditions (e.g., the slicing location along the z-axis).

We denote the set of this high dimensional mapping (Fig. 1c) as $\{{{\textbf{z}}}_{k}\}_{k=0}^{K-1}$, where *K* is the total number of observations (slices along the z-axis), and the corresponding set of measurements as $\{{{\textbf{y}}}_{k}\}_{k=0}^{K-1}$. The task is to find the optimal set of network parameters ${\varvec{\theta }}^*$ that satisfy:

1 $$\begin{aligned} {\varvec{\theta }}^{*}=\mathop {\mathrm {arg\,min}}\limits _{{\varvec{\theta }}} \frac{1}{K}\sum ^{K-1}_{k=0}\left\| {{\textbf{y}}}_{k}-{{\textbf{R}}}_{\varvec{\varphi }}( f_{{\varvec{\theta }}}({{\textbf{z}}}_{k}) ) \right\| ^{2}_{2} + \lambda \cdot {{\textbf{T}}}{{\textbf{V}}}(f_{{\varvec{\theta }}}({{\textbf{z}}}_{k})) , \end{aligned}$$

where ${{\textbf{R}}}_{\varvec{\varphi }}$ is the Radon transform^26^ with the projection lines defined by the angles ${\varvec{\varphi }}\in {{\mathbb {R}}}^N$ because the raw X-ray data come in the form of integrated projections, the ${{\textbf{T}}}{{\textbf{V}}}$ term is a total variation (TV) regularizer^20^ to further encourage the reconstruction to be denoised, and $\lambda$ is the hyperparameter that controls the influence of the TV regularizer. We then get a set of reconstructed images $\{{{\textbf{x}}}_k\} =f_{{\varvec{\theta }}^*}({{\textbf{z}}}_k)\in {{\mathbb {R}}}^{H\times W}$, where *H* and *W* are the height and width of the reconstructed image, which correspond to measurements $\{{{\textbf{y}}}_k\}\in {{\mathbb {R}}}^{N\times D}$, where *D* is the number of valid pixels in a single measurement. In our experiment, we use $N=360$, going from 0 to 179.5$^\circ$ with a step size of 0.5$^\circ$.

Note that optimization of Eq. (1) does not require clean and noise-free data as the ground truth. Instead, we find a neural network that can express the raw input data faithfully.

#### Latent space

**Figure 2:**
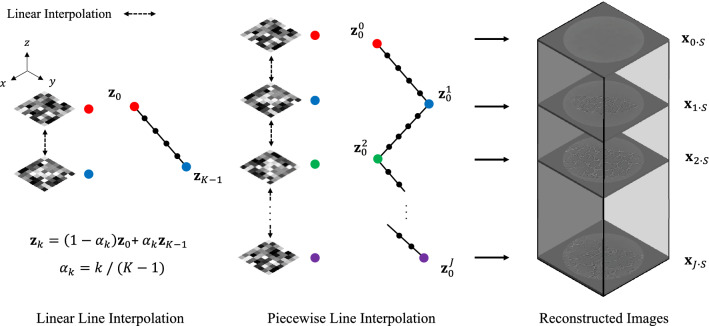
An illustration of the input latent variable interpolation and the effect of embedded data prior to improve the reconstructed image quality.

A naive implementation of this pipeline would be to consider each latent encoding for each slice ${{\textbf{z}}}_k\in {{\mathbb {R}}}^Z$ independently (we set $Z=324$), but because the problem is ill-posed, this approach does not yield good reconstruction in practice. Instead, we follow^23^ and enforce our prior knowledge that the change in structure is continuous and smooth; for this purpose, we apply a piece-wise linear mapping of the latent space (manifold); see Fig. 2. The simple latent design of a straight linear line is insufficient to embed the full context of structural information along the z-axis. Therefore, we use piece-wise linear latent variables to exploit various textures along with multiple z slices:

2 $$\begin{aligned} {\textbf{z}}_{k} = {\textbf{z}}_{S\cdot j+s} = {\textbf{z}}_{s}^j = (1-\alpha _{s})\,{\textbf{z}}^j_{0}+ \alpha _{s}\,{\textbf{z}}^{j+1}_{0}, \end{aligned}$$

where $\alpha _{s}=s/S$, $k=0\cdots K-1$ is an index for z-slice along the z-axis, $j=0\cdots J-1$ is an index for z-slice stack, and $s=0\cdots S-1$ is an index for latent variable in z-slice stack. As a result, we generate *J* stacks of latent variables, where each stack contains *S* interpolated latent variables (we set $J=32$ and $S=17$). Furthermore, the last latent variable in each stack is smoothly continuous with the first one in the following stack. Thus, we construct a set of piece-wise linear variables by stacking such stacks of latent variables.

#### Generative network architecture

**Figure 3:**
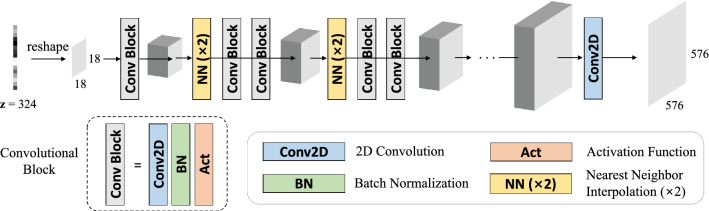
Network architecture. We repeat the intermediate blocks five times. The blue, green, orange, and yellow blocks indicate the 2D convolution layer, the batch normalization layer, the activation function, and the upsampling layer ($\times 2$), respectively.

Our generative network $f_{\theta }$ consists of convolutional layers, batch normalization layers, activation functions, and nearest neighbor upsampling layers (Fig. 3). The convolutional layers are designed to extract features that preserve the spatial dimension, whereas the upsampling layer doubles the spatial scale.

#### Radon transform

Let ${\textbf{R}}_{\varphi }\in {\mathbb {R}}^{N \times M}$ be the differentiable linear operator, which represents the cumulative effect of performing the 2D Radon transform along the lines of angle $\varphi$. Thus, the linear system for X-ray projection can be modeled as:

3 $$\begin{aligned} {\textbf{y}} = {\textbf{R}}_{\varphi }{\textbf{x}} \end{aligned}$$

where ${\textbf{x}}\in {\mathbb {R}}^{N}$ is a vector of images *x*, which has finite size $(N_{1} \times N_{2})$, and ${\textbf{y}}\in {\mathbb {R}}^{M}$ is a vector of measurements y, which has finite size $(M_{1} \times M_{2})$.

The structure includes five nearest neighbor upsampling layers, each followed by the combination of a convolutional layer, a batch normalization layer, and a ReLU activation function. The convolution layer is used in the first layer with a batch normalization layer and a ReLU activation function, whereas the convolutional layer is the only one in the last layer.

#### 3D shape reconstruction

Our method takes piece-wise linear variables as input and generates high-quality reconstructed 2D CT images. Stacking these 2D slices along the z-axis yields an entire 3D CT image. For analysis, we construct the 3D alveolar duct and alveoli by binarizing the reconstructed 3D CT image using Cellpose^27^, which is a deep-learning-based cell segmentation algorithm ;see Fig. 5a.

### Implementation details

We use an image-generation architecture^28^ that converts a 324 dimension latent code to a $576\times 576$ res. image. We sample the latent variables as $\{{\textbf{z}}^{j}_{s}\}^{j\in [0, \ldots , J-1]}_{s\in [0, \ldots , S-1]} \sim {\mathcal {U}}(0, 0.1)$ with $J=32$, $S=17$ piecewise linear components. We use Adam optimizer^29^ with an initial learning rate of $10^{-3}$ and decay the learning rate by a factor of 0.9 for every 2 k iteration. We use TorchRadon^30^ for Radon transform. We use $\lambda =10^{-2}$ for all experiments unless stated otherwise.

When run on an Nvidia Titan RTX (24 GB), our unoptimized implementation takes approximately 30 min to reconstruct one sequence of CT images or equivalently a single 3D CT image (540 2D CT images with $576\times 576$ res.) from a set of tomographic projections (360 projection images of alveoli with $540\times 576$ res.).

### Comparative methods

We compare our results with two baseline methods: (1) filtered back projection (FBP), which is a commonly used algorithm for CT reconstruction^26^ and (2) conjugate gradient for least squares (CGLS)^31^, a Krylov subspace iterative algorithm that converges faster is less sensitive to noise compared to FBP^32^. We use the implemented algorithms of FBP in^30^ and of CGLS in^32,33^.

### Evaluation metrics

We use (1) pixel accuracy, the proportion of correct predictions to total predictions, (2) Jaccard index, the size of the overlapped area divided by the size of the union area, and (3) Dice coefficient, which is similar to the Jaccard index but less penalizes the incorrect predictions, for evaluating the segmentation performance. Moreover, we evaluate the reconstruction performance using (4) peak signal-to-noise ratio (PSNR) defined as the proportion of the maximum power of a signal to the power of noise, and (5) multi-scale structural similarity (MS-SSIM)^34^, which performs as structural similarity (SSIM)^35^ at multiple scales for predicting the perceived image quality.

## Results

### Dataset

Currently, no public dataset is available for synchrotron X-ray CT reconstruction of live animals that has sufficient detail about the interior parts. Therefore, (1) we capture *a real dataset* with a synchrotron X-ray (as we described in “Synchrotron X-ray imaging”), and (2) we generate *a synthetic dataset* by using an actual alveolar structure to enable quantitative comparison of existing approaches.

#### Synchrotron X-ray projection dataset

For the validation of our idea, we follow the capturing configuration of Tracking X-ray microscopy^12^. We capture X-ray projection images of size ($540 \times 576$) that have an effective pixel size of 2.24 $\upmu$m. A total of 360 projection images of the lung apex in five live mice are taken from 0$^\circ$ to 179.5$^\circ$ at expiration. We reduce X-ray dose and unexpected movement of the lung during imaging by minimizing the number of projection images for a set of tomography.

#### Synthetic dataset

We create manually labeled binary 3D alveolar images from the noisy CT images, which are reconstructed from the real dataset, to generate the synthetic projection images ($350 \times 576$) with the same angular setting for the real dataset. We add severe Gaussian noise or speckle noise to the projection images to simulate the natural degradation that occurs during image capture. We generate this dataset because the real dataset has no ground-truth image to enable evaluation of the quality of the image reconstruction.

### Experiment with synchrotron X-ray projection dataset

**Figure 4:**
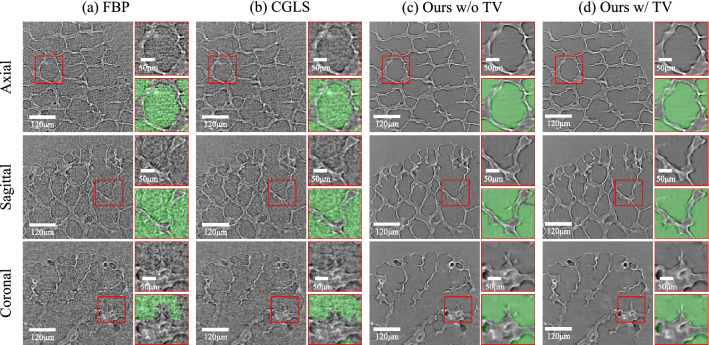
Qualitative results of synchrotron X-ray projection dataset using (**a**) FBP^26^, (**b**) CGLS^31^, (**c**,**d**) our framework without or with TV term. Red boxes indicate specific ROI of reconstructed images and segmented images.

**Table 1:** Quantitative evaluation of the segmented images for synchrotron X-ray projection dataset using Pixel accuracy [Pixel acc (%)], the Jaccard index [Jaccard (%)], and the Dice coefficient [dice (%)], and for synthetic dataset with additive Gaussian noise and additive speckle noise using PSNR and MS-SSIM.

	Real			Synthetic (Gaussian)		Synthetic (speckle)	
	Pixel Acc	Jaccard	Dice	PSNR	MS-SSIM	PSNR	MS-SSIM
FBP	87.35	64.89	78.46	11.64	0.386	25.29	0.555
CGLS	87.73	69.99	81.77	14.14	0.422	27.45	0.605
Ours (w/o TV)	90.99	75.33	85.77	31.66	0.988	32.39	0.987
Ours (w/ TV)	**91.15**	**75.70**	**86.02**	**32.91**	**0.989**	**32.41**	**0.988**

All values are means over all the 51 segmented alveoli. “Real” denotes synchrotron X-ray projection dataset and “Synthetic” denotes synthetic dataset. Gaussian, Speckle: types of noise. The highest values are in bold.

#### Imaging results

Our methods successfully reconstruct the cleaner alveolar CT images ($540\times 576\times 576$) compared with baseline methods (Table 1, Fig. 4). The performance difference is noticeable in segmentation results (Fig. 4). We compare our segmentation results with masks that are manually annotated by a skilled person (Table 1). The quantitative result using segmentation evaluation metrics demonstrates that our method has better performance.

**Figure 5:**
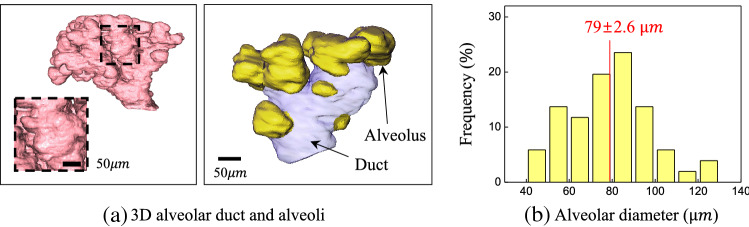
Quantitative analysis of alveolar size. (**a**) 3D images of alveolar duct and alveoli at expiration. (**b**) Alveolar diameter distribution measured in 51 alveoli from 5 intact mice at expiration.

#### Findings

We visualize the 3D morphology of alveolar ducts and alveoli (alveolar entrance on the ducts) in live mice at the expiration (demonstrated in Fig. 5a) to understand the lung’s functionality, which could be affected by alveolar shape and size^1^. High-quality 3D imaging enables us to easily distinguish between alveolar duct and alveoli even by manual process. We easily measure the size of the alveoli by determining their maximum diameters in 3D data for 51 alveoli from five mice. Alveolar diameters follow a normal distribution, but have diameters ($79\pm 2.6$ $\upmu$m (mean ± s.e.m.) at expiration (Fig. 5b) that are about twice as larger as reported in prior work that analyzes 2D images of fixed inflated lungs (about 40 $\upmu$m)^36,37^. Furthermore, in this study, the maximum value of the alveolar diameters is > 120 $\upmu$m, which is three times larger than the reports. Interestingly, recent studies for alveolar diameters measured in intact lungs, are larger than the reports for fixed lungs as 58 $\upmu$m^38^ and 40 $\upmu$m^39^ at even deflated lungs. Thus, we expect that 3D visualization of intact lungs will help to understand lung functionality and diseases more accurately.

### Experiment with the synthetic dataset

#### Comparative results

Our approach reconstructs better CT images $(350\times 576\times 576)$ than the previous methods in the presence of Gaussian noise (Table 1, Supplementary Fig. 1) and speckle noise (Table 1, Supplementary Fig. 2). Remarkably, our method successfully generates a clear alveolar structure almost identical to GT images, whereas baseline methods provide retain noise. A comparison of PSNR and MS-SSIM also demonstrates the powerful denoising effect of our work. The use of the TV term in our method contributes to a minor increase in sharpness.

### Ablation study

We conduct an ablation study on the activation function in our generative network, $f_{{\varvec{\theta }}}({{\textbf{z}}})$ (Table 2). In the analysis of the real dataset, Swish^40^ outperforms the segmentation performance of the pixel accuracy, Jaccard index, and Dice coefficient. Sine^41^ and ReLU also achieve reliable segmentation, whereas Leaky ReLU^42^ provides notably weaker results than others. In the synthetic dataset, ReLU reaches the best PSNR and MS-SSIM performance against both Gaussian noise and speckle noise. Whereas Swish achieves a strong denoising effect, Sine does not work well. Similar to the real dataset result, Leaky ReLU yields low performance.

In addition to the quantitative result, ReLU reveals a clear structure, as well as less noise (Supplementary Fig. 3). Even Sine and Swish, succeed in clarifying the structure, by noise remains. Leaky ReLU yields the best-denoised image quality but fails to represent the structure. In synthetic data experiments, ReLU outperforms the reconstruction quality of PSNR and MS-SSIM, compared to other activation functions. Furthermore, ReLU is quite robust to extreme noise, whereas the results of other activation functions leave artifacts (Supplementary Fig. 3b,c). Hence, ReLU shows the best performance considering the trade-off between segmentation performance and reconstruction quality. It is robust to severe noise occurring in the actual experimental process and improves the generative network to capture the clear structure.

**Table 2:** Ablation study on activation functions (up) and TV regularizer weights (bottom) by evaluating segmentation performance for real dataset and reconstruction performance for synthetic dataset.

	Real			Synthetic (Gaussian)		Synthetic (speckle)	
	Pixel Acc	Jaccard	Dice	PSNR	MS-SSIM	PSNR	MS-SSIM
Sine	90.06	71.19	83.02	30.28	0.983	30.40	0.983
Leaky ReLU	86.36	60.86	74.71	30.21	0.981	30.61	0.983
Swish	**91.15**	**75.70**	**86.02**	31.80	0.988	31.85	0.987
ReLU	89.94	71.54	83.03	**32.91**	**0.989**	**32.41**	**0.988**
$\lambda =0.0$	90.99	75.33	85.77	31.66	0.988	32.39	0.987
$\lambda =0.001$	90.86	73.27	84.29	31.97	0.988	32.69	0.987
$\lambda =0.01$	**91.15**	**75.70**	**86.02**	**32.91**	**0.989**	32.41	**0.988**
$\lambda =0.1$	90.50	74.95	85.54	32.77	0.988	**33.46**	**0.988**

Segmentation performance is evaluated using pixel accuracy [Pixel Acc (%)], Jaccard Index [Jaccard (%)], and dice coefficient [dice (%)] and reconstruction performance is evaluated using PSNR and MS-SSIM. “Real” denotes synchrotron X-ray projection dataset and “Synthetic” denotes synthetic dataset. Gaussian, Speckle: types of noise. The highest values are in bold.

## Conclusion and limitation

We propose a neural network-based approach for 3D reconstruction of alveolar ducts and alveoli in intact mice lungs at expiration using the synchrotron X-ray in an unsupervised way. Our network successfully represents the 3D CT images of alveolar ducts and alveoli, and it enables the measurement of 3D alveolar size at the micro-meter scale. The source code will be released in the public domain. Although we significantly improve the CT image, our approach has some limitations. First, our framework cannot work if some irregular movement occurs during the projection. This type of movement has commonly occurred when anesthesia is incomplete or cardiac motion in live mice lungs. We prevent incomplete anesthesia by instillation 2–3% of isoflurane by way of the mechanical ventilator. The apex of the lung at expiration is chosen for minimizing lung movement due to cardiac motion and respiration to apply our methods. Another limitation is the issue arising from the tilted CoR in the projection images. In CT reconstruction, the object’s rotation center should be the center of the projection image, or severe artifacts occur in the reconstructed image. Our experiment is performed at the micrometer scale, so CoR errors are unavoidable; therefore, we manually fixed the center of rotation during a preprocessing step. We are eager to solve this problem by adding network parameters in future work.

## Supplementary Information

Supplementary Information.

## Data Availability

The datasets used during the current study are not publicly available, but can be provided from the corresponding author upon reasonable request.
