# Supplementary material for: Deep 3D reconstruction of synchrotron X-ray computed tomography for intact lungs
Source: Sci Rep. 2023 Jan 31;13:1738. doi: 10.1038/s41598-023-27627-y (PMC9889716; doi:10.1038/s41598-023-27627-y)
Supplement: Supplementary file 1 — Supplementary Information. [file 41598_2023_27627_MOESM1_ESM.pdf]

Supplementary Information

A Experiment with synthetic dataset

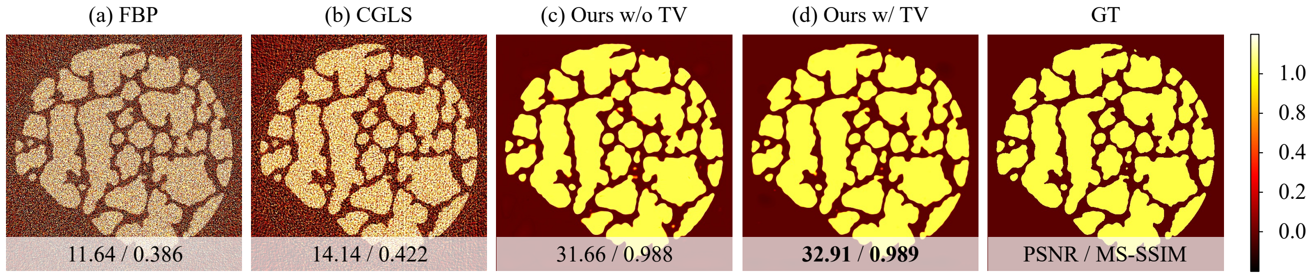

Figure 1. Comparative results (PSNR/MS-SSIM) of synthetic dataset with additive Gaussian noise. It shows an intensity map of the reconstructed images.

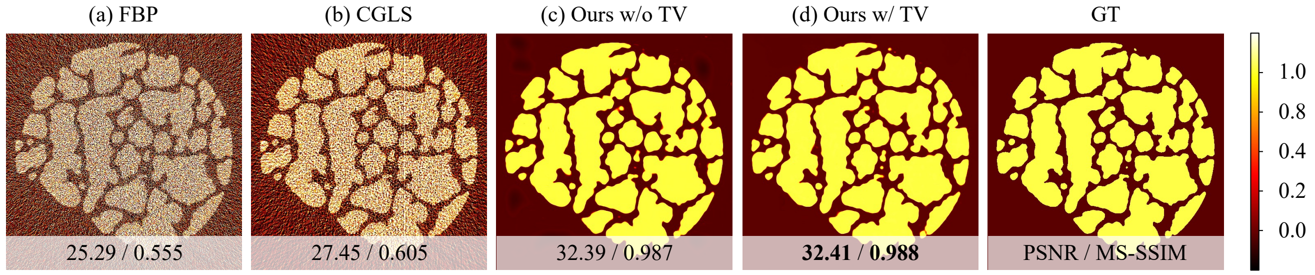

Figure 2. Comparative results (PSNR/MS-SSIM) of synthetic dataset with additive speckle noise. It shows an intensity map of the reconstructed images.

B Ablation study

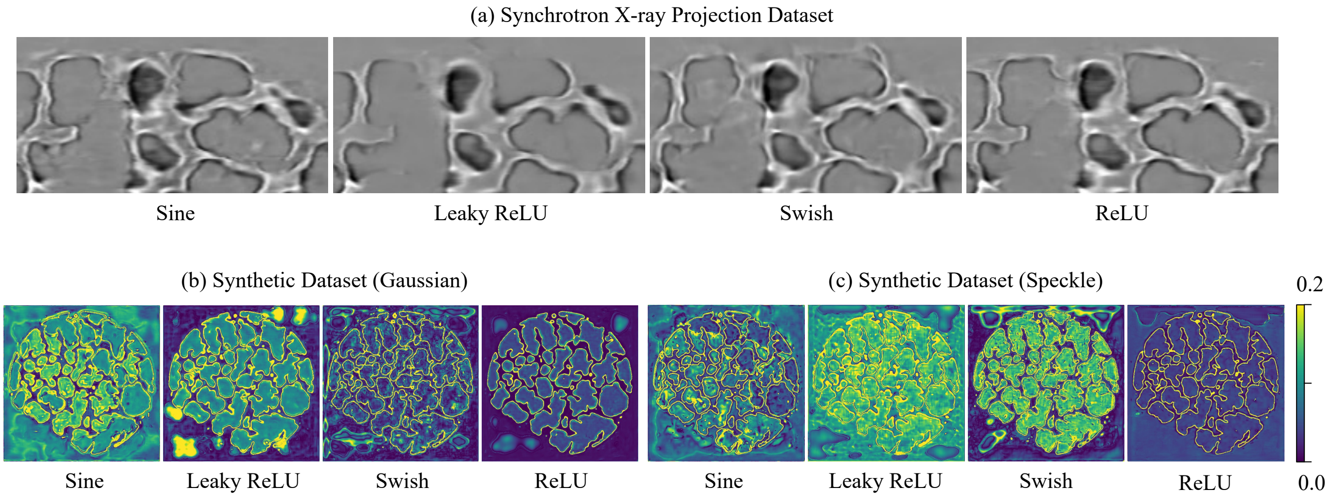

Figure 3. Ablation study on the activation functions. Qualitative comparison using (a) the synchrotron X-ray projection dataset, the synthetic dataset with (b) additive Gaussian noise, and (c) additive speckle noise. (b) and (c) show relative error map of the reconstructed images.
